# Cocoa flavanol intake improves endothelial function and Framingham Risk Score in healthy men and women: a randomised, controlled, double-masked trial: the Flaviola Health Study

**DOI:** 10.1017/S0007114515002822

**Published:** 2015-09-09

**Authors:** Roberto Sansone, Ana Rodriguez-Mateos, Jan Heuel, David Falk, Dominik Schuler, Rabea Wagstaff, Gunter G. C. Kuhnle, Jeremy P. E. Spencer, Hagen Schroeter, Marc W. Merx, Malte Kelm, Christian Heiss, for the Flaviola Consortium, European Union 7th Framework Program

**Affiliations:** 1 Division of Cardiology, Pulmonology, and Vascular Medicine, Medical Faculty, University Duesseldorf, 40225 Duesseldorf, Germany; 2 Department of Food and Nutritional Sciences, University of Reading, Reading, UK; 3 Mars Inc., McLean, VA, USA; 4 Cardiovascular Research Institute Duesseldorf, Medical Faculty, University Duesseldorf, 40225 Duesseldorf, Germany

**Keywords:** Flow-mediated dilation, Cardiovascular health, Flavanols, Blood pressure

## Abstract

Cocoa flavanol (CF) intake improves endothelial function in patients with cardiovascular risk factors and disease. We investigated the effects of CF on surrogate markers of cardiovascular health in low risk, healthy, middle-aged individuals without history, signs or symptoms of CVD. In a 1-month, open-label, one-armed pilot study, bi-daily ingestion of 450 mg of CF led to a time-dependent increase in endothelial function (measured as flow-mediated vasodilation (FMD)) that plateaued after 2 weeks. Subsequently, in a randomised, controlled, double-masked, parallel-group dietary intervention trial (Clinicaltrials.gov: NCT01799005), 100 healthy, middle-aged (35–60 years) men and women consumed either the CF-containing drink (450 mg) or a nutrient-matched CF-free control bi-daily for 1 month. The primary end point was FMD. Secondary end points included plasma lipids and blood pressure, thus enabling the calculation of Framingham Risk Scores and pulse wave velocity. At 1 month, CF increased FMD over control by 1·2 % (95 % CI 1·0, 1·4 %). CF decreased systolic and diastolic blood pressure by 4·4 mmHg (95 % CI 7·9, 0·9 mmHg) and 3·9 mmHg (95 % CI 6·7, 0·9 mmHg), pulse wave velocity by 0·4 m/s (95 % CI 0·8, 0·04 m/s), total cholesterol by 0·20 mmol/l (95 % CI 0·39, 0·01 mmol/l) and LDL-cholesterol by 0·17 mmol/l (95 % CI 0·32, 0·02 mmol/l), whereas HDL-cholesterol increased by 0·10 mmol/l (95 % CI 0·04, 0·17 mmol/l). By applying the Framingham Risk Score, CF predicted a significant lowering of 10-year risk for CHD, myocardial infarction, CVD, death from CHD and CVD. In healthy individuals, regular CF intake improved accredited cardiovascular surrogates of cardiovascular risk, demonstrating that dietary flavanols have the potential to maintain cardiovascular health even in low-risk subjects.

Flavanols, a subgroup of dietary plant-derived bioactives, have gained increasing attention, as clinical studies have shown that a higher intake of flavanol-containing foods can improve arterial function in individuals at risk for CVD and with established CVD^(^ ^1^ ^,^ ^2^ ^)^. The mechanisms of action underlying the biological effects of flavanols are not completely understood. However, flavanols are one of the few bioactives known today, for which causality between intake and an improvement in arterial function has been formally demonstrated^(^ ^3^ ^)^. A recent meta-analysis of forty-two randomised controlled human dietary intervention studies also demonstrated significant acute and chronic (up to 18 weeks) flavanol-dependent cardiovascular benefits^(^ ^4^ ^,^ ^5^ ^)^. The observed cardiovascular benefits include the recovery of endothelial function, a decrease in blood pressure (BP) and improvements in lipids and insulin resistance^(^ ^6^ ^)^. For the most part, these effects have been studied in individuals at increased cardiovascular risk, such as smokers, or those with established hypertension, diabetes, hypercholesterolaemia and manifest coronary artery disease^(^ ^4^ ^,^ ^5^ ^)^.

Whether or not the intake of cocoa flavanol (CF) has the potential to maintain and/or improve cardiovascular health in individuals without CVD and those with low degrees of cardiovascular risk, that is, healthy subjects, has not been evaluated. To yield meaningful results, studies aimed at addressing this point ideally need to be conducted in the context of broader age-range and include both sexes, and they need to be carried out for durations of 4 weeks or longer^(^ ^7^ ^)^. Thus, in the present study, we sought to investigate the effects of dietary CF intake on established surrogate markers of cardiovascular risk in healthy, middle-aged individuals without history, signs or symptoms of CVD. As the primary end point, we selected to measure endothelial function as flow-mediated dilation (FMD), and included assessments of plasma lipids, BP and pulse wave velocity (PWV) as secondary end points. Using a well-characterised CF-containing test drink and its CF-free control, we undertook a randomised, double-masked, 1-month dietary CF intervention assessing the above end points. In addition, based on applying the Framingham Risk Model we also aimed at providing an initial evaluation of the potential for dietary CF in the context of primary CVD prevention.

## Methods

### Study participants and study design

**Fig. 1:**
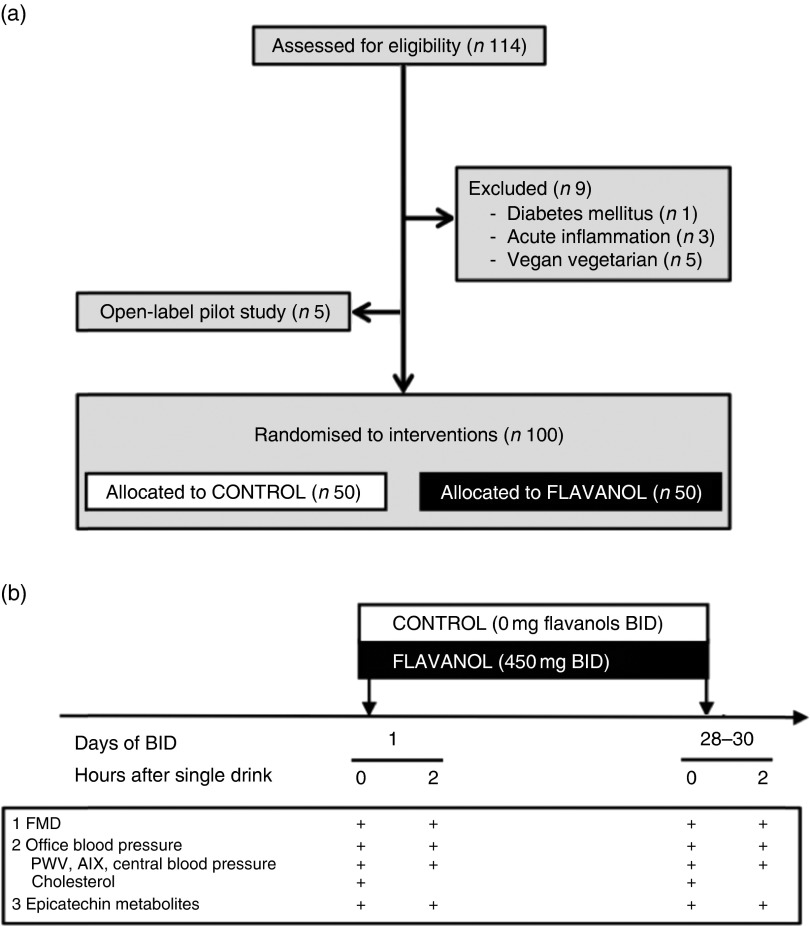
(a) Study flow (CONSORT diagram) and (b) study protocol of the randomised controlled study. BID, twice daily; FMD, flow-mediated dilation; PWV, pulse wave velocity; AIX, augmentation index.

The study took place at the Division of Cardiology, Pulmonology and Vascular Medicine of Duesseldorf University in Duesseldorf, Germany from February 2013 to August 2014. We recruited subjects by word-to-mouth and by postings at the University and University Outpatient Clinic. All subjects who came to our attention were recruited for eligibility screened and consecutively included if eligible. A total of 114 consecutive subjects underwent assessment for eligibility (see Fig. 1 for CONSORT diagram). We included 105 middle-aged (35–60 years) healthy Caucasian male and female subjects without history, signs or symptoms indicative of CVD, including previous myocardial infarction (MI), stroke and peripheral artery disease or current or previous medication, and a BMI of 23–27 kg/m^2^ (inclusion criteria). Exclusion criteria were manifest or suspected CVD, diabetes, kidney failure, acute inflammation or a heart rhythm other than sinus, extreme diets including vegetarians and vegans, alcoholism and vitamin supplement use.

We first performed an open-label kinetic pilot study in five male volunteers. The subjects received 450 mg of CF twice daily for 1 month to determine the time course of endothelial function by FMD, which measures endothelial function and is a prospectively validated surrogate marker of cardiovascular risk.

Subsequently, we performed a 1-month double-masked, randomised, controlled trial in 100 participants who were randomly (block randomisation) assigned to one of two parallel groups (1:1 ratio of intervention and controls): either the CF intervention group (FLAVANOL; 450 mg of total flavanols twice daily) or the CF-free intervention group (CONTROL; 0 mg of total flavanols twice daily). All interventions were provided as drink powder in sachets to be freshly prepared by mixing with approximately 500 ml of water. Drinks were consumed twice a d: one beverage in the morning with breakfast (06.00–08.00 h) and one with the evening meal (18.00–20.00 h). We did not require the study subjects to follow a strict timing. This regimen was maintained for 1 month, with compliance assessed by the collection of empty sachets on the last study day visit. All measurements were taken in the fasting state before (‘baseline’) and at 2 h after the first drink on day 1, and at 1 month (day 28–30) again in the fasting state and at 2 h after consumption of the last drink. Subjects were asked to refrain from consuming excess amounts of flavanol-rich foods (list provided to participants) for 24 h before study visits. No other restrictions before and during intervention regarding diet and lifestyle were required. FMD was the primary end point. Secondary pre-specified end points included office BP and plasma total, LDL and HDL-cholesterol concentration in order to determine cardiovascular risk parameters according to the Framingham Risk Prediction model^(^ ^8^ ^)^. The 10-year risks to develop CHD or CVD, to experience a MI or stroke or to die from CHD or CVD were calculated using the Framingham equation in the Excel calculator (http://cvrisk.mvm.ed.ac.uk/). Further pre-specified secondary end points included parameters derived from pulse wave analysis, including PWV, aortic augmentation index (AIX), reflective of vascular physicomechanics and central systolic blood pressure (SBP) and diastolic blood pressure (DBP). A tertiary pre-specified end point was the total plasma epicatechin metabolite concentration.

Subjects were asked to follow a 10-min supine rest period before examinations and to remain in a supine position throughout the examination, including BP measurements, FMD, pulse wave analysis and blood draws following the identical sequence. FMD was measured on the right arm, and BP measurements and blood draws were performed on the left arm.

Participants and investigators were masked throughout the study with regard to flavanol content of the test drinks, and intervention drinks were allocated in random order using block randomisation. As the open-label pilot study had only one arm, analyses were performed in an observer-blinded manner. The ethics committee of the Heinrich-Heine University approved the study protocol, and all subjects gave written informed consent (Clinicaltrials.gov: NCT01799005).

### Test materials

Both interventions used a low-energy fruit-flavoured beverage mix (provided by Mars Inc.), which was standardised and matched in composition. All beverage mixes were agglomerated powders with a maltodextrin base into which flavouring and sweeteners were incorporated. The beverage mixes were provided in sachets (7 g, equals one serving) labelled with an alphanumeric identifier to enable a double-masked study design. A high-flavanol cocoa extract (Cocoapro^®^-processed cocoa extract; Mars Inc.) was the source of flavanols in the CF-containing drink. The CF-containing drink (FLAVANOL) provided 450 mg of total CF per serving^(^ ^9^ ^)^. The total amount of CF in mg represents the sum of all monomeric flavanols and their oligomers (i.e. procyanidins) with a degree of polymerisation up to and including 10 (i.e. DP 1–10). The predominant monomeric flavanol in this drink was (−)-epicatechin (see Table 1). The control beverage mix did not contain any cocoa extract, and thus it provided 0 mg of CF (CONTROL). Given the natural presence of theobromine and caffeine in cocoa extract, both theobromine and caffeine were added to the control beverage mix in order to match the composition of alkaloids in the CF-containing test product. Colouring was also added so that the 0 mg CF CONTROL drink was also indistinguishable in appearance. Compositional details for the 0 mg (CONTROL) and 450 mg CF (FLAVANOL) test drinks are provided in Table 1.

**Table 1:** Composition of interventional vehicles ingested bi-daily

	FLAVANOL	CONTROL
Total cocoa flavanols (mg)	450	ND
Monomers (mg)	73	ND
(−)-Epicatechin (mg)	64	ND
(−)-Catechin (mg)	7	ND
(+)-Catechin (mg)	2	ND
(+)-Epicatechin (mg)	ND	ND
Dimers–decamers (mg)	377	ND
Theobromine (mg)	44	46
Caffeine (mg)	10	6
Fat (mg)	0	0
Carbohydrates (mg)	6000	6000
Protein (mg)	100	100
Energy (kJ)	104·6	104·6
Energy (kcal)	25	25
Na (mg)	3	3
K (mg)	95	85

ND, not detectable.

### Flow-mediated vasodilation and power analysis of the primary end point

FMD and nitroglycerin-mediated vasodilation (NMD) were measured in the brachial artery of the right arm using 5-min forearm occlusion by ultrasound (Vivid I; GE Healthcare) in combination with a semi-automated analysis system (Brachial Analyzer; MIA), as described^(^ ^10^ ^)^. The intra- and inter-individual variability for FMD measurements established in our laboratory are 0·9 % (sd of difference between repeated FMD measurements in twenty middle-aged healthy subjects, unpublished) and 1 % (sd within a group of healthy old subjects)^(^ ^11^ ^)^. FMD was defined as the primary outcome. On the basis of previous intervention studies with CF, we expected a change in FMD by 1·3 %^(^ ^4^ ^)^. Assuming an sd of change in FMD of 1 %, fifty experimental and fifty control subjects would provide sufficient power to detect an absolute change in FMD of 0·6 % (two-sided α of 5 %, power=0·80). As we were planning on recruiting 50 % women, we also kept the possibility in mind that women may respond differently from men. Therefore, it should be mentioned that under the same conditions twenty-five experimental and twenty-five control subjects would provide sufficient power to detect an absolute change in FMD of 0·8 % (two-sided α of 5 %, power=0·80)^(^ ^12^ ^)^.

### Blood pressure measurements

Office BP was measured using an automated clinical digital sphygmomanometer (Dynamap) at the upper left arm in supine position, after 10 min of rest in a quiet room with the arm at the heart level. We took three measurements, discarded the first and averaged the second and third for further analysis. Central BP was derived from peripheral pulse wave analysis (SphygmoCor^®^; AtCor-Medical).

### Cholesterol and clinical chemistry measurements

All clinical chemistry parameters including total, LDL and HDL-cholesterol, TAG (enzymatic photometric assay; Roche Diagnostics), Hb_A1c_, C-reactive protein (quantitative immunoturbidimetric assay; Roche Diagnostics), glucose (hexokinase assay) and whole blood count (flow cytometry; Sysmex) were measured from yellow and purple top serum vacutainer tubes using standard techniques by the Institute for Clinical Chemistry, University Duesseldorf, Germany.

### Pulse wave analysis

Central BP parameters including AIX and PWV were measured in supine subjects by applanation tonometry using the SphygmoCor^®^ system. By means of a transfer function, the pressure waveform of the ascending aorta was synthesised. PWV was determined from measurements taken at the carotid and femoral artery, as previously described^(^ ^13^ ^)^.

### Estimation of background flavanol intake

Background flavanol intake was assessed using a modified version of the EPIC Norfolk^(^ ^14^ ^)^ FFQ. Flavanol monomer, epicatechin and CF intakes were calculated using the Flaviola Flavanol Food Composition Database^(^ ^15^ ^)^.

### Analysis of flavanols and their metabolites in plasma

(−)-Epicatechin and its related metabolites were analysed in plasma by HPLC-FLD/UV and electrochemical detection using authentic standards provided by Mars Inc., as previously described^(^ ^16^ ^)^. Before analysis, plasma samples (0·5 ml) were defrosted on ice and then subjected to *β*-glucuronidase and sulfatase treatment (2000 U/ml; 40 min; 37°C). Then, samples were mixed with 2 ml of acidified ice-cold methanol (0·5 % acetic acid in methanol, v/v) containing 3′-*O*-ethyl-(−)-epicatechin (500 nm) as a recovery standard. Samples were centrifuged at 17 000 ***g*** for 15 min at 4°C and the supernatant was collected. The pellet was extracted again with 2 ml of acidified ice-cold methanol (0·5 % acetic acid in methanol, v/v) containing 3′-*O*-ethyl-(−)-epicatechin (500 nm), and then with 1 ml of 50 % methanol acidified with 0·5 % acetic acid and containing 3′-*O*-ethyl-(−)-epicatechin (500 nm). Combined supernatants underwent concentration (to approximately 50 µl) using a Speedvac system (Thermo Fisher Scientific Inc.) and were mixed with resorcinol (300 pmol) and catechol (300 pmol) before analysis by HPLC. Flavanol monomers and *O*-methylated metabolites were analysed using a Hewlett-Packard 1200 series HPLC (Hewlett-Packard) equipped with diode array and fluorescent detection. Samples (50 µl) were injected onto a reversed-phase Phenomenex Luna C18(2) column (4·6×150 mm) with 3-µm particle size. The mobile phase consisted of (A) HPLC-grade water, (B) 200 mm Na acetate, pH 4·4/methanol (84/16) and (C) acetonitrile, and the following gradient protocol was run: 0 min, 75 % A, 25 % B; 5 min, 75 % A, 25 % B; 20 min, 65 % A, 25 % B; 28 min, 63 % A, 25 % B; 34 min, 55 % A; 25 % B; 41 min, 45 % A, 25 % B; 45 min, 25 % B, 75 % C; 55 min, 25 % B, 75 % C; 56·1 min 75 % A, 25 % B; 60 min, 75 % A, 25 % B. The flow rate was 0·8 ml/min. Detection of flavanols and their metabolites was performed using a fluorescent detector with an excitation wavelength of 276 nm and an emission wavelength of 316 nm. The concentration of each compound was determined using an external calibration curve produced with the use of authentic standards.

### Statistical methods

The characteristics of the study population are expressed as mean values and standard deviations. Changes in FMD values in the pilot study were compared by one-way repeated-measures ANOVA and are presented as mean values and their standard error of means. The primary test for an effect in the RCT was an independent *t* test comparing the difference between changes due to FLAVANOL and CONTROL at 1 month. Mean values of results are presented as mean values and their standard error of means, and differences between responses are presented as mean values and 95 % confidence intervals. Responses to treatments were calculated as changes in respective parameters (e.g. FMD): 1-month values minus baseline values on day 1. We also evaluated the difference between changes at 2 h after acute consumption of intervention drinks on day 1 and at 1 month as compared with baseline on day 1. Data not normally distributed are presented as median (interquartile range), and group differences are compared with Mann–Whitney *U* tests. Analyses were computed with SPSS 20 (IBM). Correlations were presented as Pearson’s *r*.

## Results

### Baseline characteristics of the healthy study population

None of the 105 subjects had been previously diagnosed with or had clinical signs or symptoms of CVD. None of the participants were on current medical treatment, including medication, and the baseline demographic parameters were within normal limits (Table 2). There were no statistically significant differences between the CONTROL and FLAVANOL group with regard to these parameters. Furthermore, our analysis of FFQ showed that the estimated intake of flavanols was low, comparable to the general European public^(^ ^15^ ^)^, and there was no difference between the groups. On the basis of their Framingham Risk Scores, the participants exhibited a low risk – that is, the 10-year risk to be diagnosed with CHD or to experience a MI was 3·4 (sd 3·7) % and 1·3 (sd 2·1) %, respectively (*n* 105). The estimated risk to die from CVD over the next 10 years was 0·5 (sd 0·9) %.

**Table 2:** Baseline characteristics of study groups (Mean values and standard deviations)

	FLAVANOL		CONTROL		
	Mean	sd	Mean	sd	P*
*n*	55		50		
Male/female	30/25		25/25		
Age (years)	45	8	44	9	0·778
BMI (kg/m^2^)	25	3	26	3	0·310
Height (cm)	180	10	178	7	0·453
Weight (kg)	83	14	82	14	0·579
Creatinine (μmol/l)	71	9	80	18	0·991
Total cholesterol (mmol/l)	4·8	0·9	4·8	1·1	0·564
LDL-cholesterol (mmol/l)	3·0	0·9	3·1	0·9	0·315
HDL-cholesterol (mmol/l)	1·6	0·4	1·5	0·3	0·900
TAG (mmol/l)	1·3	0·4	1·1	0·5	0·104
Fasting plasma glucose (mmol/l)	5·0	0·5	4·8	0·4	0·727
Hb_A1c_ (%)	5·4	0·3	5·5	0·4	0·649
SBP (mmHg)	126	14	127	10	0·546
DBP (mmHg)	80	9	79	6	0·811
Heart rate (bpm)	64	9	64	8	0·908
CRP (mmol/l)	0·9	0·3	0·9	0·3	0·692
Hb (mg/l)	143	13	140	20	0·721
Leucocytes (1000/ml)	5·8	0·3	5·5	0·3	0·489
Flavanol monomer intake (mg/d)†	35	43	32	45	0·926
Epicatechin intake (mg/d)†	11	8	10	9	0·781
Cocoa flavanol intake (mg/d)†	106	85	106	82	0·701

CRP, C-reactive protein; DBP, office diastolic blood pressure; SBP, office systolic blood pressure. * *P* value from the unpaired *t* test. † Values are medians and interquartile ranges and were analysed using the Mann–Whitney *U* test.

**Fig. 2:**
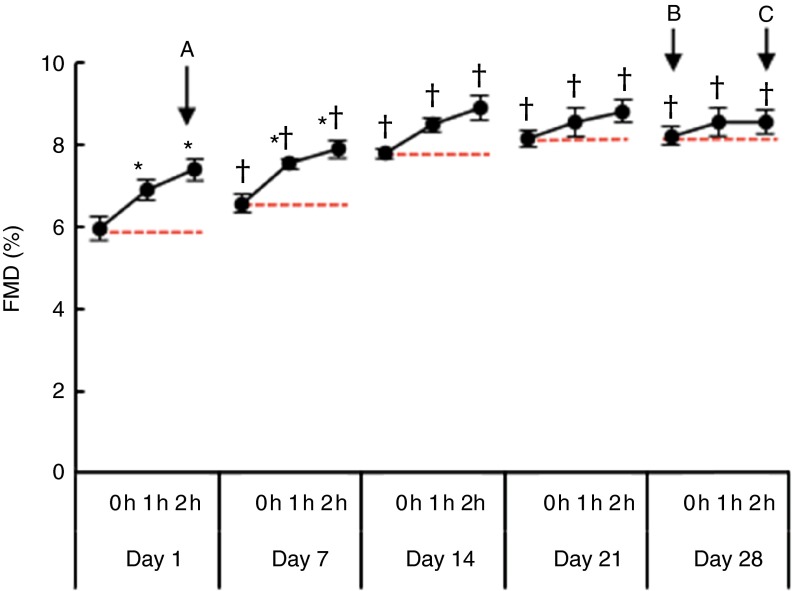
Time course of flow-mediated dilation (FMD) during the open-label pilot study. Measurements were taken before (0 h) and at 0, 1 and 2 h after ingestion of the first intervention drink on days 1, 7, 14, 21 and 28 while consuming the drink twice per d (*n* 5). Ingestion of a single test drink led to an ‘acute’ increase in FMD on days 1 and 7 (A). Although the FMD value as measured after repeated consumption of the drink increased the ‘chronic effect’ (B), ‘acute-on-chronic’ improvements were no longer statistically significant at days 14, 21 and 28 during daily consumption (C). * *P*<0·05 *v*. 0 h at the same day, respectively, † *P*<0·05 *v*. 0 h at day 1. Values are means with their standard errors represented by vertical bars.

The first five subjects recruited were male, and they participated in the open-label pilot study to (a) verify the efficacy of the flavanol drink to increase FMD, (b) allow re-assessment of effect size to power the main study and gain insight into (c) temporal responses and timing of the main study (Fig. 2). The remaining 100 subjects were included in the main study and randomly assigned to either FLAVANOL or CONTROL treatment. The test drinks were well tolerated and not a single adverse event was reported.

### Time course of FMD during daily open-label FLAVANOL consumption over 1 month

We first performed an open-label pilot study in five male volunteers, who received 450 mg of CF twice daily for 1 month to determine the time course of FMD, the primary end point of the main randomised controlled study. FMD measurements were taken in the fasting state before (0 h) and at 1 and 2 h after consumption of the first drink in the morning on days 1, 7, 14, 21 and 28.

As depicted in Fig. 2, the time course identified an ‘acute’ increase in FMD at 1 and 2 h after consumption of CF and a ‘chronic’ increase in fasting FMD. With bi-daily CF consumption over weeks, the FMD as measured after overnight fasting (>12 h after consumption of last intervention drink) increased from day 1 (6·0 (sem 0·3) %) to day 7 (6·6 (sem 0·2) %) and again from day 7 to day 14 (7·8 (sem 0·1) %). At days 21 and 28, we observed no further increase in the fasting FMD and acute ingestion, suggesting a saturation of CF-related ‘chronic’ effects (day 21: 8·2 (sem 0·2) %, day 28: 8·2 (sem 0·2) %). The change in fasting FMD between day 1 and day 28 was 2·3 (sd 0·4) %. Although fasting FMD ‘chronically’ increased, additional ‘acute’ CF consumption only significantly improved FMD at days 1 and 7.

### Flavanol intake improves endothelial function

In the 1-month randomised controlled trial, FLAVANOL consumption led to a significant improvement in FMD as compared with CONTROL (Table 3). FMD improved by 0·7 % (95 % CI 0·5, 0·9 %) at 2 h and by 1·2 % (95 % CI 1·0, 1·4 %) at 1 month. There were no differences in baseline FMD between men (5·3 (sem 0·1) %) and women (5·3 (sem 0·1) %, *P*=0·764 *t* test). The baseline diameter of the brachial artery and NMD remained unaffected by either FLAVANOL or CONTROL.

**Table 3:** Overview of primary, secondary and tertiary end points (Mean values with their standard errors; 95 % confidence intervals)

	FLAVANOL (*n*·50)								CONTROL (*n*·50)													
	Baseline		2 h		1 month		1 month, 2 h		Baseline		2 h		1 month		1 month, 2 h		Difference		Difference		Difference at	
	Mean	sem	Mean	sem	Mean	sem	Mean	sem	Mean	sem	Mean	sem	Mean	sem	Mean	sem	at 2 h*	95 % CI	at 1 month*	95 % CI	1 month, 2 h*	95 % CI
Primary end point (%)																						
FMD	5·3	0·1	6·0	0·1	6·4	0·1	6·8	0·1	5·3	0·1	5·4	0·1	5·3	0·1	5·4	0·1	0·7	0·5, 0·9†	1·2	1·0, 1·4†	1·6	1·3, 1·8†
Secondary end points (mmHg)																						
Office SBP	126	2	124	2	121	2	121	2	127	1	127	2	126	2	125	2	−1·8	−4·9, 1·2	−4·4	−7·9, −0·9†	−4·6	−7·1, −0·1†
Office DBP	79	1	77	1	75	1	76	1	78	1	78	1	78	1	79	1	−2·4	−5·1, 0·2	−3·9	−6·7, −1·1†	−3·8	−6·2, −1·3†
Total cholesterol (mmol/l)	4·84	0·13			4·64	0·13			4·79	0·13			4·77	0·10					−0·20	−0·39, −0·01†		
LDL-cholesterol (mmol/l)	3·08	0·13			2·90	0·13			3·11	0·13			3·11	0·10					−0·17	−0·32, −0·02†		
HDL-cholesterol (mmol/l)	1·53	0·05			1·61	0·05			1·50	0·05			1·48	0·05					0·10	−0·04, 0·17†		
PWV (m/s)	7·2	0·2	6·9	0·2	6·8	0·2	6·8	0·2	7·2	0·2	7·2	0·2	7·2	0·2	7·2	0·2	−0·3	−0·6, −0·1†	−0·4	−0·8, −0·04†	−0·4	−0·7, −0·005†
AIX (%)	17	2	14	2	12	2	10	2	15	2	15	2	15	2	14	2	−3·3	−6·4, −0·3†	−5·3	−8·9, −1·6†	−5·9	−9·5, −2·3†
Central SBP (mmHg)	118	2	115	2	112	2	113	2	116	2	117	2	116	2	115	2	−2·8	−5·5, −0·2†	−5·3	−8·5, −2·1†	−3·8	−6·8, −0·7†
Central DBP (mmHg)	80	1	78	1	76	1	76	1	79	1	79	1	79	1	79	1	−2·5	−4·8, −0·8†	−4·7	−7·8, −1·7†	−4·4	−7·1, −1·7†
10-year CHD risk (%)	3·4	0·5			2·7	0·4			3·4	0·5			3·4	0·5					−0·6	−1·3, −0·3†		
10-year MI risk (%)	1·3	0·3			0·9	0·2			1·3	0·3			1·3	0·3					−0·5	−0·8, −1·2†		
10-year stroke risk (%)	0·8	0·1			0·6	0·1			0·7	0·1			0·7	0·1					−0·1	−0·2, 0·03†		
10-year CVD risk (%)	4·9	0·7			3·9	0·5			4·8	0·7			4·8	0·7					−1·0	−1·7, −0·4†		
10-year CHD death risk (%)	0·4	0·1			0·2	0·1			0·4	0·1			0·4	0·1					−0·2	−0·3, −0·004†		
10-year CVD death risk (%)	0·5	0·1			0·3	0·1			0·5	0·1			0·5	0·1					−0·2	−0·3, −0·03†		
Tertiary end point (nmol/l)																						
Epicatechin metabolites	4	3	991	64	71	26	1049	76	0‡	0	0‡	0	0‡	0	0‡	0	987	857, 1118†	68	10, 125†	1045	890, 1200†

AIX, augmentation index; DBP, diastolic blood pressure; FMD, flow-mediated dilation; MI, myocardial infarction; PWV, pulse wave velocity; SBP, systolic blood pressure. * Differences with CI are from the unpaired *t* test comparing FLAVANOL with CONTROL changes from baseline. † 95 % CI does not span zero, indicating significant difference.

‡ Below limit of detection.

### Flavanol intake lowers blood pressure and decreases vascular stiffness

The consumption of FLAVANOL over 1 month led to a significant decrease in office SBP by 4·4 mmHg (95 % CI 0·9, 7·9 mmHg) and office DBP by 3·9 mmHg (95 % CI 1·1, 6·7 mmHg) as compared with CONTROL (Table 3). Pulse wave analysis showed that central SBP and DBP responses were similar to peripheral BP results with significant lowering by 4·3 mmHg (95 % CI 7·6, 0·8 mmHg) and 4·7 mmHg (95 % CI 1·7, 7·8 mmHg), respectively. PWV and AIX decreased by 0·4 m/s (95 % CI 0·8, 0·04 m/s) and 5·3 % (95 % CI 2·1, 8·5 %) at 1 month of FLAVANOL as compared with CONTROL. No changes in body weight were observed.

### Flavanol intake decreases total and LDL-cholesterol, but increases HDL-cholesterol

Both total and LDL-cholesterol significantly decreased by 0·20 mmol/l (95 % CI 0·39, 0·01 mmol/l) and 0·17 mmol/l (95 % CI 0·32, 0·02 mmol/l), whereas HDL-cholesterol increased by 0·10 mmol/l (95 % CI 0·04, 0·17 mmol/l) at 1 month after FLAVANOL as compared with CONTROL. No significant changes were observed with regard to TAG, fasting plasma glucose or Hb_A1c_.

### Plasma levels of structurally related flavanol metabolites

At the baseline visit (day 1, 0 h), the fasting levels of total plasma flavanols were below the limit of detection in ninety-eight subjects. At 2 h after acute consumption of FLAVANOL, the concentration of structurally related flavanol metabolites significantly increased in plasma (987 nmol/l (95 % CI 857, 1118 nmol/l)) over CONTROL. After 1 month of twice-daily CF consumption and following overnight fasting, fasting levels were again in all but seven below the limit of detection. Additional acute ingestion of FLAVANOL in contrast to CONTROL led to flavanol plasma concentration increase of 1045 nmol/l (95 % CI 890, 1200 nmol/l), but these levels were not significantly different from values observed after acute ingestion on day 1 (*t* test). Whereas ‘acute’ changes in FMD could be directly related to bioactive flavanol metabolites, ‘chronic’ changes in FMD as measured after overnight fasting are difficult to directly relate to plasma flavanols, as there were basically no plasma flavanols detectable in the subjects at this time point. The mechanism underlying the effects may be related as judged by the fact that there is no acute effect once a chronic effect is established after more than 2 weeks of chronic consumption but are probably different in nature as they persist after overnight fasting. However, we observed a significant correlation between an increase in FMD and increase in plasma flavanols at 2 h post consumption (*r* 0·44, *P*<0·0001) and also a significant correlation between ‘chronic’ FMD improvements at 1 month and the acute increase in plasma flavanols at 2 h as an index of individual flavanol bioavailability (*r* 0·67, *P*<0·0001).

### Initial assessments of the impact of flavanol intake on CVD risk using Framingham Risk Scores

As described above, the study participants were healthy and at low cardiovascular risk based on their Framingham Risk Scores. To evaluate the primary preventive potential of flavanols, we calculated changes in components of the Framingham Risk Score using the age, the total and HDL-cholesterol and office SBP values (absolute changes are given in Table 3). FLAVANOL led to significant decreases in the estimated 10-year risk to be diagnosed with CHD (percent decrease relative to baseline: 21 % (95 % CI 8, 33 %)) or CVD (percent decrease: 22 % (95 % CI 8, 36 %)) or experience MI (percent decrease: 31 % (95 % CI 9, 51 %)). The decrease in stroke risk was not statistically significant. The predicted 10-year risk to die from CHD or CVD was lowered (percent decrease: 37 % (95 % CI 17, 56 %) and 30 % (95 % CI 10, 49 %)) by FLAVANOL over CONTROL.

## Discussion

### Study design and effect size of *cocoa flavanol*-induced changes on vascular function

The Flaviola Health study is the first randomised controlled trial to establish the efficacy of CF for improvement of established parameters of vascular function in healthy middle-aged individuals. Several features of this study are distinctive in testing the concept that dietary CF intake can maintain vascular health: (1) the study cohort consisted of healthy individuals without any history, signs or symptoms of CVD, and it is representative for a middle-aged European population with regard to both general health status and habitual average flavanol intake^(^ ^15^ ^)^; (2) vascular function was comprehensively assessed at the level of conduit and resistance arteries, including acute and long-term effects, allowing for a comprehensive characterisation of circulatory function; (3) the study was undertaken using a randomised, double-masked design, and based on the use of nutrient-matched and standardised CF-rich and CF-free drinks, allowing for the mitigation of potentially confounding effects mediated by other bioactive food components; (4) a preceding open-label run-in study permitted reliable re-assessment of effect size and time course, enabling power analysis and final design for the main Flaviola Health study; and (5) changes in vascular function were measured along with established surrogates of cardiovascular risk, such as BP and cholesterol, allowing the assessment of changes in individual cardiovascular risk scores^(^ ^7^ ^,^ ^17^ ^)^. Taken together, this approach enabled us to test the hypothesis of whether or not CF intake improves vascular function even in healthy individuals. In addition, we aimed at assessing the effects of CF in the context of the maintenance of vascular health – a concept that gained renewed attention through recent publications by the American Heart Association^(^ ^18^ ^)^ and others – and at evaluating the potential of CF in primary CVD prevention and public health.

The primary end point of the study was FMD. FMD is a validated prognostic marker of cardiovascular risk, and an improvement in 1 % FMD has been associated with a decrease in overall CVD risk over 3·6 years of 8 %^(^ ^19^ ^)^. In the present study, in which we assessed FMD after overnight fast, we observed a significant increase in FMD of 1·2 % after 1 month of daily CF consumption. Although the 1-month duration of this study represents one of its limitations, this time frame of assessing changes in FMD is regarded as clinically relevant^(^ ^7^ ^)^, according to the recent EFSA consensus statement on the scientific requirements for studies aiming at supporting FMD-based health claims^(^ ^7^ ^)^. Together with the fact that this study is the first larger-scale flavanol dietary intervention to investigate healthy populations, we would advance that our data are pertinent and meaningful. Generally considering the effect size in the context of previous interventions with FMD as the primary end point, which do not thus far include longer-term studies in healthy individuals, similar degrees of improvement have been reported for some, but not all, interventions in patients^(^ ^20^ ^)^, including the use of ACE inhibitors^(^ ^21^ ^)^, statins^(^ ^20^ ^,^ ^22^ ^)^, as well as various lifestyle modifications, including physical exercise training^(^ ^23^ ^)^. Taken together, our study suggests that the endothelial functional improvements because of CF intake are similar in effect size, or even greater, compared with established primary and secondary preventive strategies^(^ ^20^ ^–^ ^22^ ^)^.

### *Cocoa flavanol* intake decreases blood pressure, vascular stiffness and cholesterol

Throughout middle and old age, BP is strongly and directly related to cardiovascular (and overall) mortality, without any evidence of a threshold down to at least 115/75 mmHg^(^ ^24^ ^)^. Dividing people into ‘hypertensives’ and ‘normotensives’ is common practice, but it is problematic as there are now sufficient trial data to show a statistically significant risk reduction when decreasing the BP even in ‘normotensive’ individuals, without known vascular disease^(^ ^25^ ^)^. In this context, the risk reduction is rather due to the decrease in BP *per se*, and there seems to be little or no difference between commonly used BP-lowering medications for primary prevention of CVD^(^ ^26^ ^)^. The results in the present study show that CF intake led to a significant decrease in SBP and DBP in healthy individuals. This decrease in BP was detected in central and peripheral arteries. The effect size in our current study (a decrease of in-office BP of 4·4/3·9 mmHg (SBP/DBP)) was comparable to that described in a meta-analysis (comparison of CF intake with true CF-free controls using a blinded study design in patients with CVD or subjects being at risk for CVD (3·7/2·7 mmHg)^(^ ^5^ ^,^ ^27^ ^)^) and approaches the BP-lowering effect sizes observed by typical BP-lowering medications^(^ ^28^ ^)^.

So far, no other study of this scale has addressed whether or not flavanols can decrease BP in healthy subjects with normal BP at the beginning of the study. The parallel changes in PWV and aortic augmentation are consistent with the notion that an improved elasticity of arteries or arterial unloading may be related to SBP lowering. The strong association between ageing and increases in PWV as a marker for vascular stiffness supports that PWV is a marker of vascular age^(^ ^29^ ^)^. In our study, the PWV significantly decreased toward values that are usually seen in persons several years younger. The parallel changes in central BP and AIX suggest that this may have positive effects on cardiac function. Future studies will have to verify the biological relevance of such findings in the context of longer-term investigations and characterise the mechanisms involved. In the context of the present study, we cannot mechanistically dissect whether or not the CF intake-induced change in PWV may be primarily caused by a softening of the aorta and large vessels or are secondary to alterations in cardiac stroke volume, changes in central and peripheral BP or because of alterations in the total peripheral resistance. (We have addressed the effect of CF on haemodynamics in much more detail in a recent study^(^ ^30^ ^)^.)

In middle- and old age, and at all BP levels, high plasma cholesterol is positively associated with IHD mortality^(^ ^31^ ^)^. Depending on age, a decrease in total cholesterol of 1 mmol/l was associated in middle-aged individuals with a decreased risk of IHD mortality by 50 %. We here show in healthy volunteers that CF may be able to decrease total and LDL-cholesterol by 0·2 mmol/l over 1 month. A meta-analysis investigating the effects of cocoa products on lipids in diverse populations suggested an overall total and LDL-cholesterol-lowering effect of 0·2 mmol/l but no significant effect on HDL-cholesterol^(^ ^32^ ^)^. Overall, these outcomes were confirmed by a more recent meta-analysis including fourteen studies^(^ ^4^ ^)^. The results differed from the first meta-analysis^(^ ^32^ ^)^ in so far as they demonstrated a small, but significant, increase in HDL-cholesterol in longer-term studies^(^ ^4^ ^)^, which is consistent with our current study, thus supporting that CF may be capable of improving blood lipids in healthy volunteers.

### Primary prevention, cardiovascular health and dietary guidelines

By applying the Framingham framework of risk assessment to our data set, it can be demonstrated that our study population was indeed healthy and at low risk for CHD (which is defined as a 10-year risk to develop CHD of <10 %, compared with 3·4 % in the present study). More importantly, it can be estimated that CF intake has the potential to reduce the 10-year risk of CV morbidity and mortality even in this healthy cohort by 20–30 % and 30–40 % (percent relative to baseline value), respectively. Naturally, such estimates would be based on the assumption that outcomes in terms of efficacy and safety would persist over years of chronic CF consumption. Thus, although the application of Framingham risk scoring in this context should be interpreted with some caution, as our study was limited with regard to scope and duration, the outcomes of this assessment provide a useful basis for initial assessments of the potential of CF in the context of primary CVD prevention, and for data interpretations and discussions in the light of potential future studies.

### Conclusion

The outcomes of the Flaviola Health study, a CF-based dietary intervention trial in healthy middle-aged men and women, demonstrate improvements in accredited surrogates of cardiovascular risk, including indices of vascular function and metabolism. Our findings support the notion that CF intake has the potential to support the maintenance of cardiovascular health. Furthermore, our data add to the accumulating body of evidence regarding the health benefits of dietary flavanols and procyanidins in general, thus contributing to evidence-based assessments of potential future dietary guidelines for these bioactives.
